# Heterotopic Pancreas as a Leading Point of Intussusception: A Case Report

**DOI:** 10.30699/IJP.14.2.180

**Published:** 2019-06-10

**Authors:** Hiva Saffar, Seyed Mohammad Tavangar, Salma Sefidbakht, Roghayyeh Aghapour, Fatemeh Molavi

**Affiliations:** 1 *Associate professor, Department of Pathology, Shariati Hospital, Tehran University of Medical Sciences, Tehran, Iran*; 2 *Professor, Department of Pathology, Shariati Hospital, Tehran University of Medical Sciences, Tehran, Iran*; 3 *Assistant professor, Department of Pathology, Shariati Hospital, Tehran University of Medical Sciences, Tehran, Iran*; 4 *Anatomical and clinical pathology resident, Department of Pathology, Shariati Hospital, Tehran University of Medical Sciences, Tehran, Iran*; 5 *Internal medicine resident, Department of Internal Medicine, Shariati Hospital, Tehran University of Medical Sciences, Tehran, Iran*

**Keywords:** Intussusception, Heterotopic, Pancreas

## Abstract

Heterotopic pancreas (HP) is generally asymptomatic and found incidentally. It can act very rarely as a leading point for intussusception. Thus, it should be considered as a differential diagnosis of the mass lesions leading to the intestinal intussusception. Herein, we report an unusual case of HP as a cause of ileocolic intussusception.

## Introduction

Heterotopic pancreas (HP) also known as ectopic, aberrant accessory pancreas (1) is defined as the presence of pancreatic tissue outside its usual or habitual location lacking anatomical or vascular continuity to the main pancreas (1-5).

The incidence of ectopic pancreas varies between 0.5-3.7% which can be found at any position in the abdominal cavity (1), however, it is more common in the upper gastrointestinal (GI) tract including stomach, duodenum and jejunum (2, 3). The ileal lesions are much less common (4).

Despite rather common occurrence of HP, they are generally asymptomatic and found incidentally (1). They may become clinically symptomatic when are complicated by inflammation, bleeding, obstruction or malignant transformation (1, 5).

They may stand as a leading point for intussusception very rarely (1). The definite diagnosis depends on the histopathologic examination (1).

Herein, we are going to describe an unusual case of HP leading to the intestinal intussusception who was suffering from recurrent episodes of abdominal pain and melena.

## Case Report

A 24-year-old male with the past medical history of peptic ulcer disease and on and off episodes of melena since two years ago, referred to the Emergency Department after twelve hour of severe periumbilical abdominal pain, nausea and vomiting. He stated that he had been admitted for one day with chief complains of burning epigastric abdominal pain one week ago. Physical examination revealed right upper quadrant abdominal tenderness with no rebound tenderness. Additional review of the system was unremarkable.

Distal part of ileum was irregular and thickened with areas of ulceration in small bowel series which had been performed two years earlier.

In the present admission, except for the low hemoglobin level (12.4 g/dl), other laboratory tests results were within normal limits. Ultrasonography was performed and revealed a short segment of bowel pulled into dilated loop of intestine with a target sign appearance suggestive for intussusception. The spiral CT scan of abdomen also demonstrated ileocolic intussusception.

**Figure 1:**
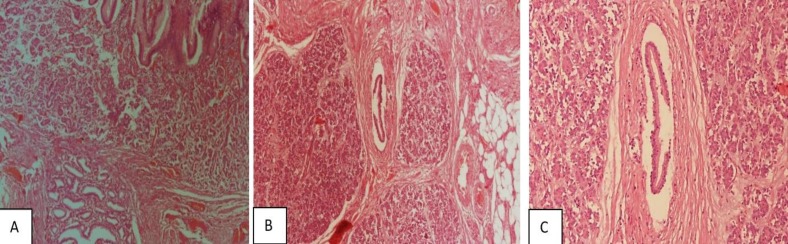
(A): Presence of pancreatic ducts and acini in mucosa and Submucosa of intestine. (B): Extension of the pancreatic tissue deep to sub serosa. (C): Pancreatic lobules and ducts in higher magnification

The patient was sent to the operation room. The diagnosis of ileocolic intussusception was confirmed. Right hemicolectomy was performed. On gross exam, the leading point was a submucosal polypoid lesion with extensive hemorrhage and ulceration measuring 2.5 cm in its greatest dimension. The sectioning of the mass revealed yellowish discoloration. Microscopic evaluation showed ectopic pancreatic tissue mainly located in submucosa extending deep to sub serosa with focal mucosal involvement (Figure 1). In table 1 a summary of some recently reported cases of HP leading to intestinal intussusception is demonstrated.

**Table 1:** A summary of the cases of HP leading to intestinal intussusception

No	Age(Y/O)	Gender	Sign/symptoms	location	Reference
1	47	male	On/Off episodes of melena and constipation with abdominal pain	Terminal ileum	
2	72	male	Acute onset of nausea/vomiting and abdominal pain	Ileocecal junction	
3	24	male	Epigastric pain and vomiting	Proximal jejunum	
4	24	female	Nausea/intermittent abdominal cramping pain	jejunum	
5	14	male	Fever/severe abdominal pain	ileum	
6	7	female	Intermittent cramping abdominal pain	ileum	
7	12	male	Paroxysmal colicky abdominal pain with non-bilious vomiting	ileum	
8	22	female	Severe abdominal pain with bile-stained vomiting (pregnant)	ileum	
9	9	male	Repeated episodes of colicky intense periumbilical pain with bilious vomiting	ileum	
10	39	male	Recurrent left abdominal discomfort	jejunum	
11	12	male	Severe abdominal pain with intermittent vomiting	ileum	
12	1(15 months)	male	severe colicky abdominal pain and repeated bilious vomiting/bloody stool	ileum	

## Discussion

The incidence of localized pathological leading point for intussusception varies from 2% to 12% in large series (1). Isolated HP is usually asymptomatic but can act as a leading point for intussusception (1). 

There are several theories explaining the occurrence of HP. One theory believes that during the embryonic rotation of the dorsal and ventral buds, fragments of the pancreas become separated and deposited at ectopic sites (1).

HP can be seen through the whole GI tract but more common cases have been reported from stomach (25-38%), duodenum (17-36%), and jejunum (15-21%) (1). The involvement of other sites including ileum is very rare (1).

As mentioned earlier, HP is usually asymptomatic and identified incidentally during surgery for other conditions (1) or rarely can act as a leading point for intussusception (1).

The preoperative diagnosis is difficult. The symptomatic patients usually refer with vague abdominal pain and signs of obstruction (2). Nausea, vomiting and GI bleeding have also been reported. The present case referred for the abdominal pain and intermittent episodes of melena, which the latter was the same as the case reported by Ahmed Monier et al (1).

It is believed that symptomatic lesions are generally more than 1.5 cm and located adjacent to or directly involved in mucosa (3, 6).

Macroscopically, HP mostly appears as yellow nodule most commonly located in the submucosa with the least common serosal surface (3).

Our specimen showed submucosal lesion measuring 2.5 cm in the greatest dimensions with the areas of gross hemorrhage. In microscopic evaluation, the pancreatic lobules, ducts and acini were noted.

It should be mentioned that in adults, intussusception is rare and predisposing factors should be excluded (3). Therefore, to evaluate the possible causing condition, segmental resection is recommended in adults without prior attempt to reduction (3).

Finally, albeit being rare, HP should be considered as a differential diagnosis of intestinal mass lesions (7) which could act as a leading point in intestinal intussusception.
